# Medical Legislature and Ethical Changes

**Published:** 1881-02

**Authors:** 


### Editorial.

### MEDICAL LEGISLATION AND ETHICAL CHANGES.

We notice through the various exchanges a growing tendency in the medical profession to direct attention to the correction of some of the many abuses and disadvantages under which it labors.

In New York a move in the right direction is being made to establish the "doctor in the school room," and to repeal the law that allows the establishment of medical colleges *ad libitum* in the present easy manner" (*Medical Record*). The question of procuring bodies for dissection is creating no small amount of excitement throughout the country, and demagogues are fattening upon the occasion presented of ingratiating themselves upon a superstitious public prejudice. The prejudice is simply an exponent of public ignorance and susceptibility to demagogism! We are opposed to indiscriminate exercise of the practice, but the attempt to entirely suppress it will not only be attended with failure, but much unnecessary trouble and publicity.

On the question of certain points of ethics, there is growing discontent in various sections. As the arrangement now stands the restriction acts exert themselves upon the young and uninfluential members of the profession in a mandatory manner, yet leaves the "bigger bugs" comparatively untrammelled. Restriction of the balance of power in this respect is imperatively demanded, and must come, too, sooner or later.

